# A multifunctional structural coloured electronic skin monitoring body motion and temperature†

**DOI:** 10.1039/d2sm01503j

**Published:** 2022-12-28

**Authors:** Arne A. F. Froyen, Albert P. H. J. Schenning

**Affiliations:** a Stimuli-responsive Functional Materials and Devices, Department of Chemical Engineering and Chemistry, Eindhoven University of Technology P.O. Box 513 Eindhoven 5600 MB The Netherlands a.p.h.j.schenning@tue.nl; b Institute for Complex Molecular Systems, Eindhoven University of Technology Den Dolech 2 Eindhoven 5600 MB The Netherlands; c SCNU-TUE Joint Laboratory of Device Integrated Responsive Materials (DIRM), South China Normal University, Guangzhou Higher Education Mega Center Guangzhou 510006 China

## Abstract

Multifunctional e-skins provide information on physiological and environmental parameters. However, the development and fabrication of such devices is challenging. Here, structural coloured electronic skins are presented, which are prepared *via* scalable methods that can simultaneously monitor the skin temperature and body motion when patched onto the human skin.

## Abstract 2

A structural coloured e-skin is reported, which is prepared *via* scalable methods that can simultaneously monitor the skin temperature and body motion when patched onto the human skin.

## Graphical abstract

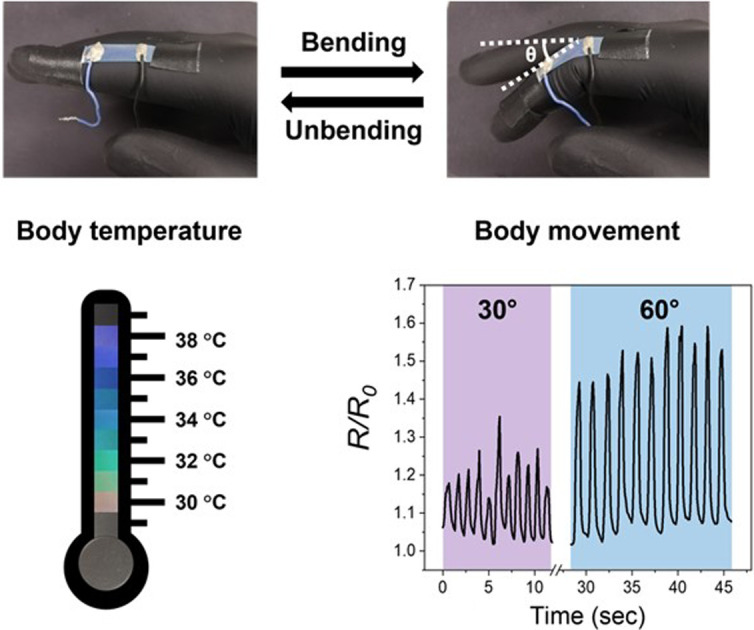

Continuously monitoring the subject's physical activity could lead to early diagnosis of illness, chronic diseases, or physiological malfunctioning, and could minimize the amount of periodically scheduled medical check-ups. As such, wearable sensors have become more abundant in our daily life; however, miniaturization and improved device integration is required to enhance user comfort.^1,2^ To realize real-time monitoring of physiological parameters with minimal user awareness, flexible human-interactive electronic skins (e-skins) are exploited, gathering data from the environment *via* sensing modules that are patched as thin films onto human skin.^3–6^ Most commonly, an electronic readout is generated for such wearables based on transduction mechanisms including piezoresistivity, piezoelectricity, and capacitance, converting an alteration of the experienced stimulus into a changing electrical signal.

To promote the widespread use of e-skins, it would be beneficial if the user can check his/her vital signs without using sophisticated machinery, for example, integrating an optical device could offer a facile readout.^7^ Although displays have been considered, these optical devices are bulky, rather expensive, and could cause adhesion and recyclability issues.^8,9^ To realize optical feedback, cholesteric liquid crystals (CLCs) can be of interest as these molecules exhibit structural colour due to their self-assembled helical structure.^10–12^ When experiencing an external stimulus, the helical periodicity can be altered corresponding to a shifted reflective colour. As such, structural coloured e-skins have been developed, displaying both optical and electrical sensing *via* a change in colour and resistance upon exerted strain, providing both qualitative and quantitative information about the degree of stretching.^13,14^

Rather than studying a single physiological parameter, more accurate and faster detection of health issues could be acquired when monitoring multiple parameters/conditions; recording the skin temperature is of particular interest as it may indicate possible illness or injuries. Therefore, multifunctional electronic skins are pursued that simultaneously monitor diverse stimuli and/or vital signs.^15^ Despite the recent realization of multimodal photonic wearables, discriminating the mechanical strain and temperature remains challenging as superimposed optical responses are inevitable while operating.^16–18^ The current state-of-the-art structural coloured e-skins are based on temperature-responsive photonic elastomers, inducing a simultaneous mechanochromic and thermochromic response.^16,17^ Thus, the creation of user-interactive photonic e-skins featuring optical feedback without disturbing the real-time electrical signal monitoring is still anticipated. Additionally, the fabrication of structural coloured e-skins *via* scalable methods remains challenging. For example, the fabrication of such films *via* roll-to-roll methods could result in a high production throughput and low-cost fabrication, hence such techniques are beneficial for upscaling.^19,20^

Here, we report multifunctional structural coloured e-skins, developed by using scalable solution-processed methods, that are capable of monitoring skin temperature and body motion *via* a respective optical and electrical response. The multimodal photonic wearables were realized by equipping a temperature-responsive, free-standing photonic film with a thin, flexible conductive layer (see ESI†). While a silver nanowire (AgNW)-based ink is used to establish an electrical output, photonic emulsions were prepared to form a free-standing structural coloured film (Fig. 1a).

**Fig. 1:**
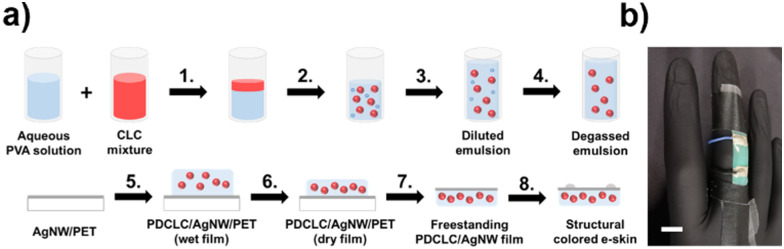
(a) Schematic overview of the development of the structural coloured e-skins. (b) Image of the multifunctional structural coloured e-skin, patched on top of a finger, visualizing the measured skin temperature (*T* = 33 °C, scale bar = 1 cm).

First, a temperature-responsive polymer dispersed cholesteric liquid crystal (PDCLC) film was developed from CLC-in-water emulsions. A thermosensitive CLC mixture (E7/S811), exhibiting a smectic-cholesteric phase transition, was formulated to promote large reflection band tuning within a small temperature interval corresponding to the human skin temperature range. After adding the CLC mixture to an aqueous polyvinyl alcohol (PVA)/glycerol solution in a 20/80 ratio (step 1), emulsification was conducted *via* shear mixing (step 2) to induce CLC droplet formation.^21–23^ Whereas PVA is frequently utilized for biomedical purposes due to its beneficial biocompatibility,^24,25^ glycerol acts as a plasticizer in the final free-standing PDCLC film, improving the e-skin's stretchability.

Afterward, the CLC microdroplet concentration was decreased to improve the photonic and mechanical properties of the free-standing PDCLC films. The undiluted, dried PDCLC films showed significant photonic cross-communication, visualized as scattering due to the close-packing of CLC microdroplets, established during film formation (Fig. S1, ESI†).^26–28^ These free-standing films easily ruptured upon stretching due to the high load of CLC microdroplets, acting as defects for crack initiation during stretching. To resolve these issues, a compatible PVA/glycerol solution was added to the shear-mixed emulsion in a 90/10 ratio to reduce the microdroplet concentration (step 3). After mixing, the diluted emulsion was degassed under vacuum (step 4) to prevent defect formation inside the PDCLC film upon drying, since air bubbles trapped in the deposited suspension caused crater evolution at the surface during film formation (Fig. S2, ESI†).

The degassed suspension was drop cast (step 5) on top of a AgNW/PET substrate, prepared by gravure printing a conductive AgNW-based ink (see ESI†).^29^ After solvent evaporation (step 6), the dried polymer film was peeled off the PET substrate (step 7), obtaining the free-standing PDCLC/AgNW film. In the final stage, conductive epoxy glue and wires were attached to the photonic film to induce proper electrical contact between the power source and the device (step 8). The photonic films, having an average thickness of 80 μm, can be patched on top of the human body and function as multimodal structural coloured e-skins thereby visualizing the skin temperature *via* a thermo-optical response (Fig. 1b). In this study, we employ such photonic wearables at room temperature, however, using these systems in more extreme weather conditions may require an adjustment of the CLC mixture to ensure proper reflection band shifting (Fig. S3, ESI†).

Gradually heating the photonic wearable resulted in a blue shift over the entire visible spectrum within a small temperature range when exceeding the smectic-cholesteric phase transition point (*T* > 29 °C) of the thermosensitive CLC mixture (Fig. 2a and b).^21,29,30^ Easily distinguishable colours were observed for temperature changes per 1 °C, hence these photonic wearables are suitable for the detection of subtle body temperature variations *via* optical feedback.

**Fig. 2:**
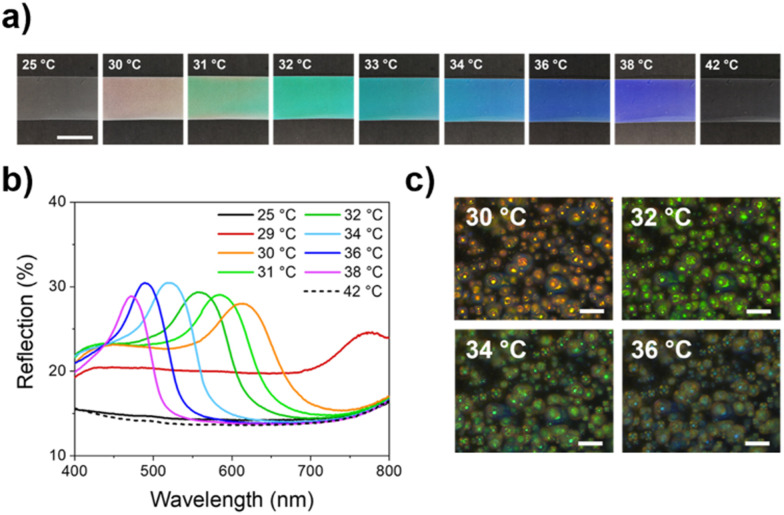
(a) Images of the temperature-responsive structural coloured e-skin demonstrating colour tuning in a small temperature interval (scale bar = 1 cm). (b) Reflection spectra of the free-standing PDCLC/AgNW film upon heating. (c) POM images (crossed polarizers) of the structural coloured e-skin showing the temperature-responsive colour shift inside the spherical CLC microdroplets (scale bar = 50 μm).

Furthermore, the reflective colour disappeared at *T* > 38 °C (isotropic phase), making these structural coloured e-skins suitable for the detection of fever, corresponding to a transparent, non-reflective e-skin. When the body temperature decreases again, the reflective colour reappears as the thermochromic response of the emulsified PDCLC systems is reversible (Video S1, ESI†).^21^

Polarized optical microscopy (POM) revealed randomly dispersed CLC microdroplets (average droplet diameter = 16 μm) trapped throughout the polymer matrix (Fig. 2c). As only spot reflection in the droplet's centre is observed, spherical rather than oblate droplets were present after solvent evaporation.^31–33^ Due to the dilution of the photonic emulsion, the droplet–droplet distance increased compared to PDCLC films obtained from the undiluted, shear-mixed emulsion (Fig. S1, ESI†), causing a reduction of the photonic cross-communication.^27,28^

The PDCLC/AgNW film exhibited angular-independent structural colour, induced by the spherical droplet shape which provided radial CLC alignment (Fig. S4, ESI†). Therefore, a uniform colouration of the e-skin was observed upon finger bending; the observed reflective colour was independent of the viewing direction, which facilitated optical readout (Fig. 3a). The wearable's colour indicates that a mechanochromic response is absent upon finger bending, suggesting that the reflection band is not affected when exerting mechanical stress. To verify this assumption, the structural coloured e-skin was stretched to different strain values (*ε* = (*l* − *l*_0_)/*l*_0_), with *ε* representing the strain, *l* the length of the e-skin upon stretching, and *l*_0_ the initial length of the photonic wearable. While stretching the film at a fixed temperature, the thermally induced reflective colour remained constant even at high strain values (Fig. 3b). Moreover, the initial shape was recovered after releasing the applied stress (*ε*(rel) = 0), demonstrating the stretchability of the wearable for the strain regime *ε* = 0–1.

**Fig. 3:**
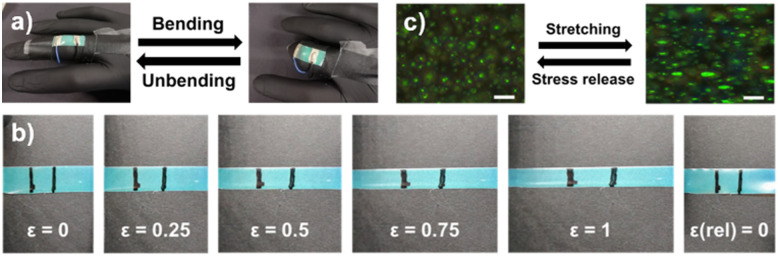
(a) Uniform, angular-independent structural colour observed upon finger (un)bending (*T* = 33 °C). (b) Photographs of the photonic e-skin (*T* = 33 °C) displaying a constant reflective colour upon stretching (*ε* = 0–1) and stress release (*ε*(rel) = 0). (c) POM images of the flexible PDCLC/AgNW film upon stretching and stress release, presenting a reversible spherical-to-oblate droplet deformation (scale bar = 50 μm).

The origin of the absent mechanochromic response was studied by analysing the influence of stretching on the PDCLC morphology and the encapsulated CLC microdroplets (Fig. 3c). During stretching, the flexible PDCLC/AgNW film elongated along the stretching direction, causing an analogous unidirectional deformation of the CLC microdroplets. The exerted strain caused a spherical-to-oblate droplet transition, thereby converting the spherical point defect into an ellipse defect.^32,34^ Although the reflection area inside the CLC droplet increased with strain,^32^ which slightly improved the film's reflection intensity, the observed reflective colour remained constant (Fig. S5, ESI†). Upon stress release, a reversible shape deformation was attained due to the soft polymer matrix, allowing recovery of the spherical droplet shape and the corresponding central reflection spot.

Apart from visualizing the skin temperature, quantitative information regarding the user's body motion could be gathered since the photonic e-skin functioned as a resistive-type strain sensor, converting the experienced perturbations/movement into an electrical signal. When mechanical stress was exerted on the wearable, the resistance of the conductive AgNW-based layer was altered: during external stretching/releasing cycles, the resistance of the structural coloured e-skin continuously changed due to the film's shape deformation, allowing for electrical signal monitoring. The main driving force for the varying electrical properties of the PDCLC/AgNW films is the disconnection of the nanowires upon unidirectional stretching, reducing the overlapping area of the nanowires and increasing the tunnelling resistance, which correspondingly decreases the film conductivity.^35–37^

While applying pressure to the e-skin with a plastic rod, the generated strain caused an increase of measured resistance (*R*) until the load was removed again, visualized by a recovery of the initial state and a decrease of *R* until the initial shape and resistance (*R*_0_) were regained (Fig. 4a, b and Fig. S6, ESI†). The time-dependent resistance plot revealed that an analogous response could be obtained during repetitive pressing cycles (Fig. 4b), validating that the conductive layer is not damaged when applying pressure to the PDCLC/AgNW film. In addition, the pressing time and amount of load applied to the system could be analysed since these parameters can be derived from the slope and height of the corresponding resistance changes. Likewise, the recovery time can be studied from the time difference between the stress removal and regaining of *R*_0_.

**Fig. 4:**
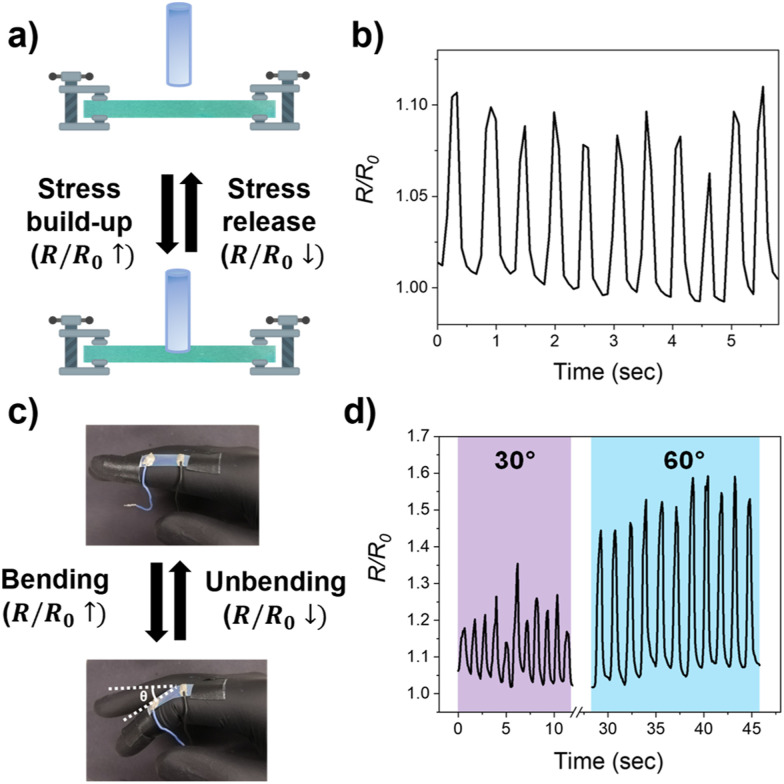
(a) Schematic representation of the fixed structural coloured wearable and the corresponding resistance change when applying pressure to the PDCLC/AgNW film with a plastic rod. (b) Electrical signal monitoring displayed a reversible resistance change when repetitively applying pressure to the structural coloured e-skin. (c) Demonstration of the multifunctional structural coloured e-skin patched to the index finger. Simultaneous detection of changes to the skin temperature or body motion could be established by the PDCLC/AgNW film. (d) Real-time resistance monitoring of the multifunctional structural coloured e-skin during cyclic finger bending for varying bending angles (*θ* = 30 or 60°).

At last, simultaneously monitoring the skin temperature and body motion was presented by attaching the structural coloured e-skin to a finger (Fig. 4c). While a temperature-responsive, angular-independent structural colour was attained that matches the skin temperature, cyclic finger bending/unbending could be detected and analysed *via* real-time resistance monitoring (Fig. 4d). Although the profile of the electrical response remained analogous for different bending angles (*θ*), the magnitude of the corresponding resistance change differed depending on the degree of bending. When increasing the bending angle, the amplitude of the resistance changes increased due to larger mechanical stress exerted on the photonic e-skin. Subsequent finger unbending resulted in a resistance drop to its initial value as the initial size of the photonic wearable is recovered, hence cyclic body motions could be monitored over time *via* an electronic readout (Fig. S7, ESI†).

## Conclusions

A multifunctional structural coloured e-skin has been developed, demonstrating simultaneous monitoring of skin temperature and body motion *via* a respective optical and electrical output. The fabrication method presented for producing the photonic wearables allows for high throughput production since scalable techniques (shear mixing and gravure printing) were used. A free-standing, temperature-responsive PDCLC film was equipped with an integrated conductive AgNW-based film. After emulsifying the thermosensitive CLC mixture and subsequent film formation, reflection band shifting was attained within the body temperature range, resulting in optical feedback in case of subtle temperature changes. A uniform, angular-independent reflective colour was observed at elevated temperature when stretching the film, without indications of a mechanochromic response, thereby differentiating itself from the currently reported structural coloured e-skins.^16,17^ Moreover, the AgNW-based layer could provide quantitative information regarding body motion, visualized as a change in the initial resistance when applying pressure or upon finger bending/unbending. The amount of mechanical stress exerted on the system could be derived from the amplitude of the resistance change while the perturbation and film recovery time could be analysed from the corresponding slopes. Apart from this example, these multifunctional structural coloured e-skin could be employed to simultaneously monitor multiple physiological parameters when patched to the human body. Such multimodal photonic wearables show promise for integration into biomedical devices to monitor various physiological or environmental parameters *via* facile feedback mechanisms.

## Conflicts of interest

There are no conflicts to declare.

## Supplementary Material

SM-019-D2SM01503J-s001

SM-019-D2SM01503J-s002
