# Supplementary material for: A multifunctional structural coloured electronic skin monitoring body motion and temperature
Source: Soft Matter. 2022 Dec 28;19(3):361–5. doi: 10.1039/d2sm01503j (PMC9846708; doi:10.1039/d2sm01503j)
Supplement: SM-019-D2SM01503J-s001 [file SM-019-D2SM01503J-s001.pdf]

Electronic Supplementary Material (ESI) for Soft Matter.
This journal is © The Royal Society of Chemistry 2022

ELECTRONIC SUPPLEMENTARY INFORMATION

A multifunctional structural coloured electronic skin monitoring body motion and temperature

Arne A. F. Froyen[1,2], Albert P. H. J. Schenning[1,2,3,]*

[1] Stimuli-responsive Functional Materials and Devices, Department of Chemical Engineering and Chemistry, Eindhoven University of Technology, P.O. Box 513, 5600 MB, Eindhoven, The Netherlands

[2] Institute for Complex Molecular Systems, Eindhoven University of Technology, Den Dolech 2, 5600 MB, Eindhoven, The Netherlands

[3] SCNU-TUE Joint Laboratory of Device Integrated Responsive Materials (DIRM), South China Normal University, Guangzhou Higher Education Mega Center, 510006, Guangzhou, China

[*]Corresponding author, email: A.P.H.J.Schenning@tue.nl

1. Development of the multifunctional structural coloured e-skins

1.1 Chemicals

Polyvinyl alcohol (M_w = 9.000-10.000 g/mol, 80 % hydrolysed, and M_w = 31.000-50.000 g/mol, 87-89 % hydrolysed) and glycerol were purchased from Sigma-Aldrich. (*S*)-octan-2-yl 4-(4-(hexyloxy)benzoyloxy)benzoate (S811) and the nematic liquid crystalline mixture E7 were obtained from TCI Chemicals and Merck, respectively. A silver nanowire containing ink (TranDuctive N15) was purchased from GenesInk.

1.2 Fabrication of the AgNW/PET substrates

The procedure to fabricate the AgNW/PET substrates with gravure printing is identical to earlier reported work.[1] A silver nanowire containing ink (TranDuctive N15, Genesink) was applied on top of a biaxially oriented transparent PET substrate having a thickness of 100 µm (Melinex 506, DuPont Teijin Films) via gravure printing (IGT F1 Printability Tester). The AgNW/PET foils were prepared by using a 'gravure printing with pre-inking' mode with a printing speed of 0.5 m/s, an anilox force of 250 N, 50 % anilox speed, and three pre-ink revolutions for the anilox before the AgNW-based ink was applied on top of the PET substrate. Afterward, the transparent foils (24 x 5 cm^2) were cured for 5 min at 90 °C in an oven to induce film formation. After curing, a four-probe method was used to determine the sheet resistance (R_s) of the printed AgNW/PET substrates: R_s = 25.97 ± 0.41 Ω sq^{-1}.

1.3 Preparation of the temperature-responsive photonic emulsion

Polyvinyl alcohol (15 wt %; M_w = 9.000-10.000 g/mol, 80 % hydrolysed) and glycerol (2 wt %) were dissolved in demineralized water while stirring at 70 °C until a homogeneous mixture was obtained. A temperature-responsive CLC mixture consisting of a nematic liquid crystalline mixture E7 (70 wt %) and a chiral dopant S811 (30 wt %) was obtained by blending these components at 60 °C to yield a homogeneous mixture. The CLC mixture was added to the aqueous PVA solution in a 20/80 weight ratio and subsequently emulsified at 35 °C for 0.5 h using an overhead stirrer (IKA T 18 digital ULTRA-TURRAX) at a stirring speed of 3000 rpm.

1.4 Fabrication of the multifunctional structural coloured e-skins (PDCLC/AgNW films)

The above-described photonic emulsion, consisting of CLC microdroplets dispersed in an aqueous PVA solution, was diluted by adding a PVA/glycerol solution (15 wt % PVA; Mw = 31.000-50.000 g/mol, 87-89 % hydrolysed, and 2 wt % glycerol in H_2O) in a 10/90 ratio, respectively. The mixture was shaken by hand at room temperature for 2 min to ensure homogeneous mixing and was degassed under vacuum before usage. The diluted emulsion was manually drop cast on top of the gravure printed AgNW/PET substrates after which the system was left to dry for at least 12 hours. After drying, smaller pieces were cut from the PDCLC/AgNW/PET foils to ensure proper contact with the human finger. The dried film was peeled off the substrate using a razor blade, yielding the temperature-responsive PDCLC/AgNW films having an average thickness of 80 µm. Before recording the resistance changes of the PDCLC/AgNW wearable, wires were attached to the photonic wearable using a conductive epoxy glue (Chemtronics, CW2400) spread out over the width of the films and cured at room temperature for 4 h to establish proper electrical contact. The wires were connected to a DC power supply (Keithley 2400 SourceMeter), which monitored the resistance of the PDCLC/AgNW film over time.

2. Characterization of the PDCLC/AgNW films

The temperature-responsive reflection band shifting was analysed by heating the free-standing PDCLC/AgNW films with an external hot plate while recording the reflection spectra with a Perkin Elmer Lambda 750 UV/vis/NIR spectrophotometer. Air was used as a baseline for the reflection measurements. Polarized optical microscopy (Leica DM2700M microscope) images were collected in reflection mode under crossed polarizers.

3. Optical properties of the dried PDCLC/AgNW films prepared from undiluted emulsions

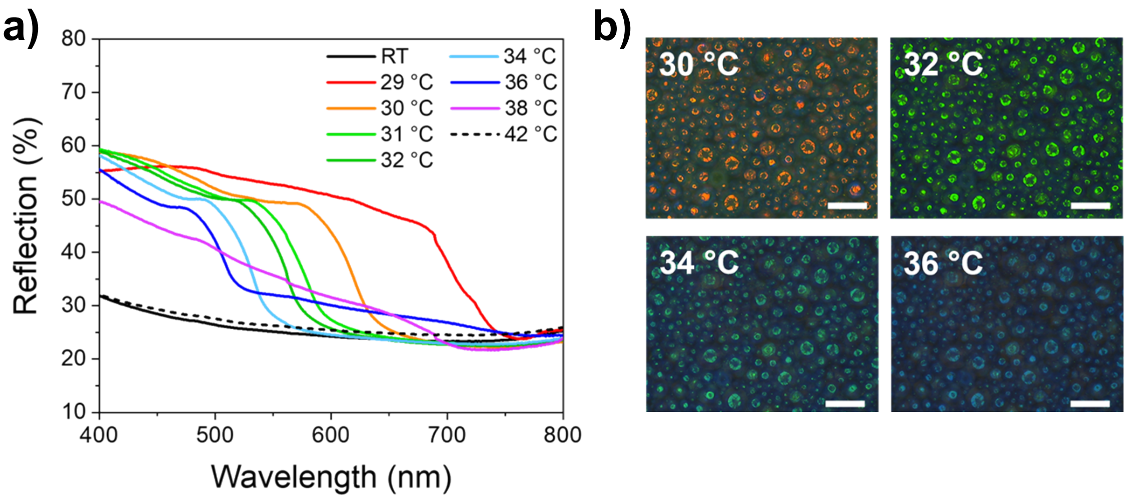

Fig. S1. a) Temperature-responsive reflection spectra of the free-standing PDCLC/AgNW film prepared from the undiluted photonic emulsion. b) POM images (measured with crossed polarizers) of the dried structural coloured e-skin prepared from the undiluted photonic emulsion, showing the temperature-responsive colour shift and photonic cross-communication inside the spherical CLC microdroplets (scale bar = 50 μm).

4. Images of the dried PDCLC/AgNW films with or without the degassing step

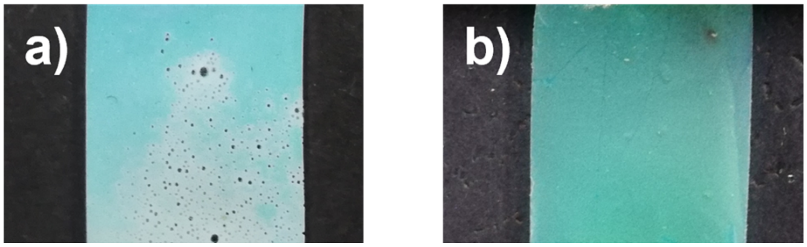

Fig. S2. Images of the dried PDCLC/AgNW film (T = 33 °C) prepared without (a) or with degassing (b) the diluted photonic emulsions before drop casting.

5. Images of the reflective PDCLC/AgNW films for different environmental temperatures

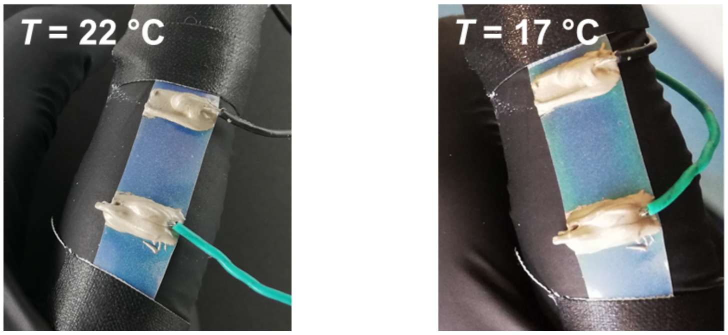

Figure S3. Temperature-responsive reflective color of the photonic wearable at different environmental temperatures. It can be observed that in this case a small color shift was observed for an environmental change of $\Delta T = 5$°C due to heat convection between the user and the environment.

6. Images of the angular-independent structural coloured e-skins

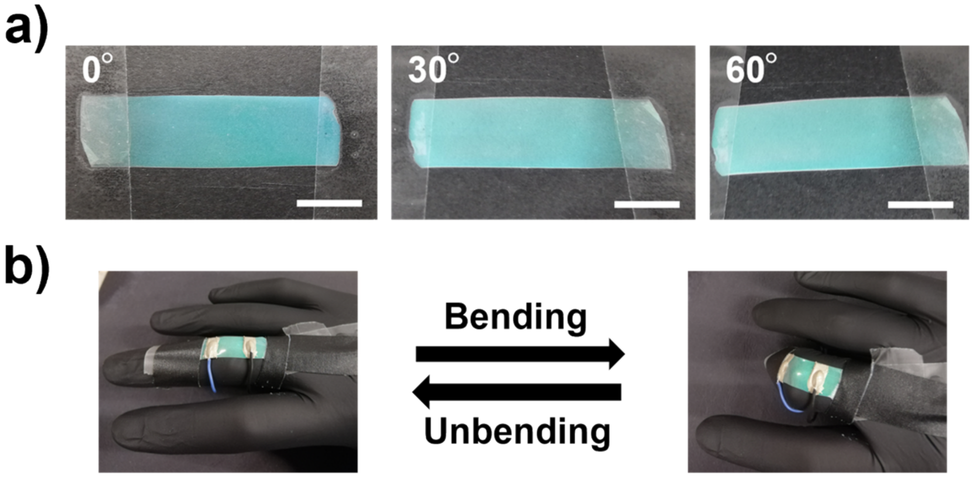

Fig. S4. a) Images of the structural coloured e-skin (T = 33 °C) captured from different viewing angles, displaying the angular-independent reflective colour. b) Uniform colouration of the structural coloured e-skin was observed upon finger bending regardless of the viewing direction (T = 33 °C).

7. Reflection spectra of uni- and bidirectionally stretched PDCLC/AgNW films

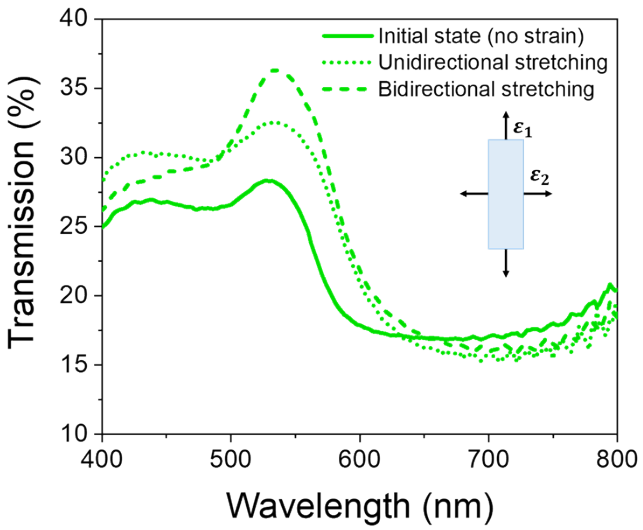

Fig. S5. Reflection spectra of a PDCLC/AgNW film (T= 33 °C) measured in the unstrained state (ε_1 = 0, ε_2 = 0) and under unidirectional (ε_1 = 0.5, ε_2 = 0) and bidirectional stretching (ε_1 = 0.5, ε_2 = 0.5).

8. Electrical signal monitoring during periodic finger bending/unbending

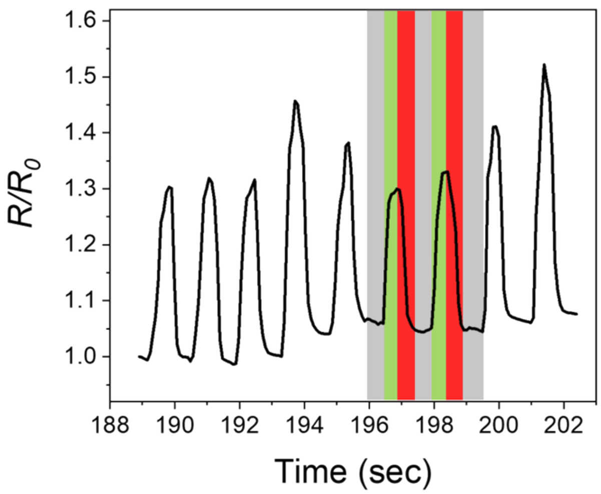

Figure S6. Real-time resistance monitoring at room temperature of the multifunctional structural coloured e-skin that experienced cyclic finger bending (green), unbending (red), and steady-state periods (grey) corresponding to time intervals for which the finger was in the rest state.

9. Response of the multifunctional structural coloured e-skins during a cyclic load

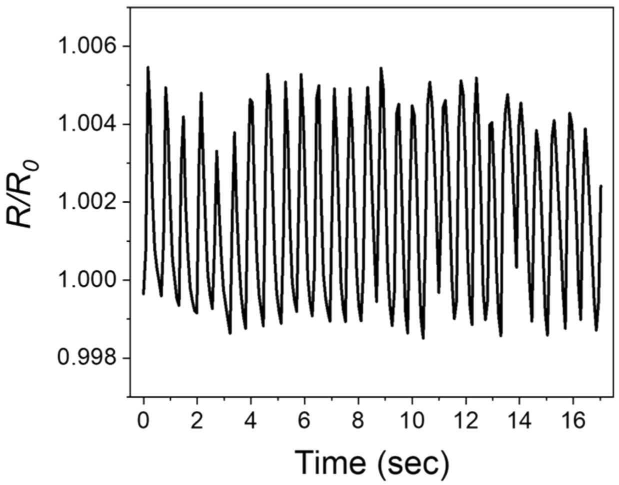

Figure S7. Real-time resistance monitoring of an e-skin that experienced tactile sensing by rapidly pressing the fixed film.

10. References (ESI)

1 A. A. F. Froyen, N. Grossiord, J. de Heer, T. Meerman, L. Yang, J. Lub and A. P. H. J. Schenning, *ACS Appl. Mater. Interfaces*, 2022, **14**, 39375–39383.
